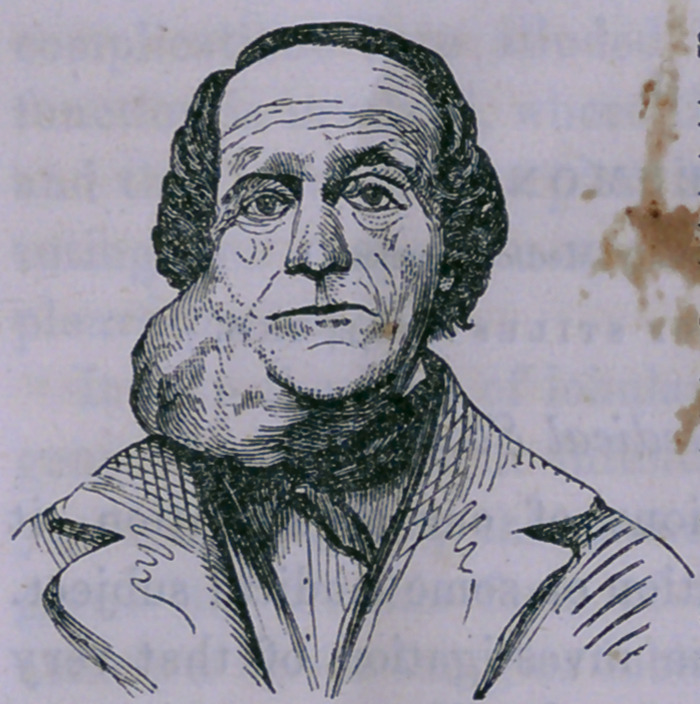# Cases of Extirpation of the Parotid Gland and Other Glandular Tumors

**Published:** 1859-12

**Authors:** Daniel Brainard

**Affiliations:** Professor of Surgery in Rush Medical College, Surgeon of the U. S. Marine Hospital, of the City Hospital, Chicago, etc.


THE CHICAGO
### MEDICAL JOURNAL.

VOL. II. DECEMBER, 1859. No. 12.

### ORIGINAL COMMUNICATIONS.

ARTICLE I.

### CASES OF EXTIRPATION OF THE PAROTID GLAND AND OTHER GLANDULAR TUMORS.

BY DANIEL BRAINARD, M.D.,

Professor of Surgery in Rush Medical College, Surgeon of the U. S. Marine Hospital, of the City Hospital, Chicago, etc.

The possibility of removing the whole of the parotid gland by operation is hardly called in question by intelligent surgeons. Indeed, it is, at the present time, difficult to understand how it should have ever given rise to the discussions which have been witnessed on the subject. Might not the possibility of removing the whole of the thyroid body, or of lower jaw, be denied with equal reason.

Allan Burns seems to have been the first to attempt to prove its removal impossible. His reason was that where the gland and all its ducts were fully injected with mercury, it could not be dissected out; the mercury escaping at numerous points. Without having made the experiment, I can easily believe that this is so. But as is remarked by Vidal, the ducts may be opened, yet the gland removed. Besides, the mercurial injection, by filling all the gland, enlarges it, and renders the pressure on the walls of the fissure containing it much greater than in the dead or living subject not injected. Some who were forced to admit the removal of the gland, still contend that it has been done rarely, and deny that most of the cases reported as such refer to the gland at all. Velpeau, in his Operative Surgery, and Beclard, in his thesis, seem to cast doubt upon all those cases in which no great hemorrhage occurred, and in which the face was not paralyzed, or in which the movements of the muscles of the face were restored after having been lost. Yet, in many of the cases whose validity is not disputed, there was little hemorrhage and no ligatures applied; and in his report to the Imperial Academy of Medicine, Oct. 26, 1858, M. Malgaigne admits that "in certain exceptional cases, on account of anomalies shown by dissections, the parotid gland may be completely removed without wounding the external carotid artery or the trunk of the facial nerve." This conclusion was sustained by the Academy. M. Naegale, as quoted by Velpeau, affirms that in the dead subject the gland may be dissected away without wounding the trunk of the nerve, and that he has removed it in the living without causing paralysis.

For myself, I should look with suspicion upon all cases where the external carotid artery is not divided, and those where the face is not paralyzed; but am sure that when the gland is attacked from below, as in McClellan's cases, this artery is often torn across and does not bleed; and I see no reason to doubt that the development of the gland may, in many cases, diminish the size, or even obliterate the artery by pressure, as happened in a case of Heyfelder. It seems certain that the hemorrhage from removing enlarged lymphatic glands is quite as great as that from extirpation of the parotid. I am not prepared to say, in regard to the restoration of the movements of the face, how far this may be due to the action of other nerves with which it is known the muscles of the face are supplied. Mr. Fergusson says that in a case in which he divided the facial nerve, the paralysis became, after a time, much less conspicuous than at first.

The entire removal of the gland has been proved by dissection, for surgeons have unfortunately lost their patients and made post-mortem examinations, as happened to Lisfranc and Beclard. Where the body, angle, ramus and condyle of the jaw have been removed, and the surface of the mastoid process, the glenoid cavity and tubercle of the temporal bone scraped, as performed by me in a case published in the *Amer. Jour. of the Medical Sciences* for Oct., 1853, there is no room for doubt.

I have since repeated this operation twice, and it was once performed by Mr. Hysern, a Spanish surgeon, as quoted by Velpeau.* When the jaw is removed, there can be no difficulty in discerning the tissues in the wound, and detecting any portions of the gland which may remain. But this is not necessary to establish the truth of the reports of the operation. If A. Cooper, Beclard, Larrey, Warren, Mott, McClellan, and the scores of surgeons who, knowing the controversy on the subject, have reported operations of the kind, are not to be credited, it will be difficult to find any reliable authority in surgery. More than this, I believe there is no reasonable ground to doubt that Heister really removed it. Any one who will consult his work, "Institutions de Chirurgie," vol. 3, p. 129, *et seq.* (1770), will find that he describes an operation in which a pound of blood is lost during the incisions, in which death often occurred, and that he criticises Gurengeot who had spoken of it as not dangerous. Heister also says that there were surgeons in his day who denied the possibility of removing the gland.

The case of Jane Sharp, as reported by John Bell, could hardly have been less than the entire extirpation of the organ. He says, "After the operation, I put my finger into the hollow whence the gland was extracted, which I felt to be two inches and a half deep at its lower angle behind the corner of the jaw bone; the carotid lay bare, beating strongly, not dilated; the upper part of the wound was deep, so that the finger touched the pterygoid process forwards and the apophysis cuneiformis backwards; and when she swallowed, the morsel, in passing down the pharynx, pressed upon the point of the finger."†

The late Professor George McClellan, who performed this operation eleven times, used to state in his lectures that the operation is much more easily performed on the living than on

---

* Operative Surgery, French edition, vol. 1, Supplementary Appendix. This part is mutilated in the translation by Townsend.

† Principles of Surgery, p. 507.

the dead subject, giving as a reason, that the gland being situated in a triangular fissure, whose base is directed outwardly, any considerable enlargement must cause it to rise out of its bed, and draw with it all those prolongations whose removal in a healthy state would be so difficult.

The same fact was noticed by Dr. Randolph and Dr. N. R. Smith, who even say that it becomes pediculated. My own experience fully confirms the views of Dr. McClellan, without, however, going so far in this respect as that of Dr. Smith.

It were easy for me to quote above fifty operations of what I deem undoubted cases of removal of the parotid gland, of which about one-half have been reported in this country, and this without including any anterior to that of Beclard, but it would be of little interest. I shall, therefore, give a report of two cases in which the gland was removed, together with some others in which operations much more difficult were performed for the removal of diseased lymphatic glands. I reserve three cases, in which the organ in question was removed along with the half of the lower jaw (disarticulated), for a subsequent communication.

I have added figures of some of the tumors, taken by daguerreotype, believing they would be useful in aiding the diagnosis, for I have generally noticed that each organ in its morbid growth assumes a form peculiar to itself, and believe that careful attention will enable the surgeon to distinguish between enlargements of the parotid and those of the glands situated in contact with it, by the form and situation alone.

CASE 1.—*Removal of the Parotid Gland for Scirrhus—Cure.*

Rebecca Dearsdorff, aged thirty years, of full habit and good health, consulted me, January 30, 1857, on account of a tumor situated between the ramus of the jaw and the lobe of the ear. The attention of the patient was first directed to this tumor four years previously, when only a slight enlargement existed. From that time it increased slowly and without any pain until within three months, when it has grown rapidly, and now presents the appearance well represented in the foregoing figure. The tumor at this time is firm to the touch and presents some inequalities; the skin over it is not discolored.

*Operation.*—The patient having been placed under the influence of chloroform, a semi-circular incision was made, commencing behind the lobe of the ear, ending upon the middle of the cheek, and passing over the lower part of the gland. The covering having been dissected up from below, the finger was thrust beneath the tumor, by which, with an occasional touch with the knife, it was readily separated from its attachments.

A circumstance especially deserving of notice is, that *the fissure in which the gland is naturally situated was nearly empty, and the finger passed into it without difficulty.*

During the operation, the portio dura was divided; the external carotid artery was torn accross, and the end of it lay in the lower part of the wound, three-fourths of an inch in length, where it had been drawn out of the tumor. It did not bleed, but, as a precaution, it was tied. The face was instantly drawn to the opposite side on the division of the nerve.

On examination of the cavity, the ramus of the jaw, the mastoid process, the styloid process in its whole length, the stylo-maxillary ligament, the auditory passage, and the ligament of the temporo-maxillary articulation, were fully exposed. At the bottom, the internal carotid artery and the internal jugular vein were distinctly seen and felt. There was no great hemorrhage. The wound was filled with a sponge until this had entirely ceased, when careful search was made by the surgeons and assistants for any remains of the parotid gland. None could be found; all the spaces into which it is said to prolong itself were vacant. The wound was dressed in the usual way, and a full dose of morphine administered.

On examining the tumor, the structure of a salivary gland was distinctly to be discerned on its entire surface. The trunk of the portio dura passed through it. The central part seemed to me very decidedly scirrhus.

The operation did not occupy ten minutes, and was by no means very difficult. The reason is that during its growth the disease had gradually raised the gland from its bed in a manner which will be readily understood. The patient recovered, and was able to return home in two weeks.

CASE 2.—S. S. Millard, aged thirty-five years, consulted me, Nov. 10, 1858, on account of a tumor situated below the left ear. He stated that he had first perceived it about two years previously, and some months since an attempt to extirpate had been made, but the surgeon, finding it deeper and more difficult to remove than he anticipated, desisted, after having cut deeply into it, and contented himself with applying cupping glasses over its surface, by which a small part of its contents had been forced out. The wound cicatrized slowly, and at the time of examination, an irregular surface was presented, red, elastic, free from tenderness and pain. The general health was pretty good.

This tumor was removed, Nov. 13, 1858, at the U. S. Marine Hospital, in presence of the class and attendants. Owing to the cicatrix upon the surface, a piece of the skin was removed. I succeeded in getting under it at the lower part and raised it out of its bed by the fingers. There was considerable hemorrhage. The external carotid, the temporal and internal maxillary arteries requiring ligatures; the two latter on account of the retrograde circulation. The pharynx, internal carotid artery, internal jugular vein, the pterygoid muscles, were distinctly felt and seen.

The patient recovered without accident, and, Oct. 16, 1859, writes that "my face is apparently well."

On examination of the tumor, it was found to be of a narrow-like appearance, with masses firmer and apparently more fibrous than the rest. The facial nerve passed through it. (The side of the face was paralyzed after the operation.) Surrounding the morbid growth, small portions of the parotid gland, in a healthy state, were noticed.

The character of the growth would have left me in doubt as to the malignancy; but the cicatrization after the first incision, and it having remained well for eleven months, leads me to think that it was not cancerous.

In this case, the lymphatic glands removed with the tumor were healthy, and the disease seemed evidently to have originated in the parotid. I therefore took great pains to search for any parts which might have remained, and a small piece upon the side of the face was detected and removed after the principal growth had been taken out.

CASE 3.—*Myeloid Tumor, situated above the Parotid Gland—Operation—Cure.*

John Halbon, of Lodi, Kane Co., Ill., consulted me, Jan. 11, 1859, on account of a tumor situated on the left parotid region, the size and form of which are represented by the annexed figure, from a daguerreotype. This had been discovered about a year previously, and its growth had been without pain or any alteration of the health. The skin over it was not discolored. It was elastic and free from tenderness.

It was removed the same day. A cyst surrounded it, which seemed to be composed of condensed cellular tissue, and the tumor itself appeared to have originated in a lymphatic gland. It extended deeply behind the angle of the jaw, but was dissected out mostly by the finger without division of any important artery. The space left was not like that found after removal of the parotid gland. The patient recovered without accident, and up to the present time there has been no return of the disease.

The tumor, on being laid open, showed a structure somewhat jelly-like, but firmer, of a reddish gray color. I think it to be what is described by Lebert as "fibro-plastic," and by Paget as "myeloid."

CASE 4.—*Enlargement of the Lymphatic Glands in the Parotid Region—Extirpation.*

The following case is presented as an example of those enlargements of the lymphatic glands said to be often mistaken for disease of the parotid gland.

S. S., aged twenty years, consulted me, May 11, 1859, on account of a tumefaction of the left side of the neck. On examination, a glandular enlargement was detected, situated behind and below the angle of the jaw. The appearance is well represented by the annexed figure.

The tumor was firm, lobulated, but did not give the sensation of scirrhus. The skin over it was, at the most prominent parts, somewhat discolored.

*History.*—The tumefaction had existed over six months, and had been treated but in a very insufficient manner with iodine. The patient's health, in other respects, was good.

March 11. The tumor was extirpated. It extended deeply down upon the sheath of the great tissues of the neck, under the angle of the jaw, and encroached upon the situation of the parotid gland. It was adherent on all sides, and was dissected out with difficulty, and a part was so invested with the great tissues, that it could not be removed. Three large arteries required ligatures, viz., the facial, lingual, and the trunk of the external carotid behind the jaw. After the operation was finished, the parotid gland was found in its natural situation, but so much scooped out that it was perhaps possible to have supposed that part of it had been removed.

The tumor, on examination, presented no appearance of cancerous tissue, but was probably of the kind called fibro-plastic.

The patient was put upon the free use of iodide potass internally, and lint placed in the wound so as to prolong the suppurative action. He did well, and returned home at the end of three weeks in a favorable state.

CASE 5.—*Cancer of the Lymphatic Glands of the Side of the Neck—Operation.*

M. N., aged forty-eight years, consulted me, Sept. 1, 1857, on account of a large tumor of the left side of the neck. The appearance of this tumor at the time is so perfectly well represented in the adjoining figure that no further description is needed. It was, as may be noticed, nearly half as large as the head, the skin discolored at the most projecting points, and veins ramifying over the surface. There was a sensation of elasticity which almost gave the idea of fluctuation. The patient was emaciated, and had that appearance of sallowness which is so generally observed in malignant disease.

*History.*—The growth of this tumor had been slow. It was first noticed about two years previously, and remained for over a year indolent and free from pain. Recently, the growth has been very active and the pain excessively severe, particularly in the right hand, arm and shoulder. It was this pain which induced me to give way to the solicitations of the patient and perform an operation for its removal. The well-marked appearances of malignancy, and the constitutional symptoms, rendered the return of the disease certain, and the size and situation of the tumor rendered the success of an operation doubtful. Many patients reported to have died of air entering the veins, or from the effect of chloroform, have undoubtedly sunk from the extent of the wound and the depressing effect of the operation.

The operation was performed, Sept. 20, 1857, at DeKalb Centre, in presence of the physicians of the place and Dr. Winer, my assistant. Nothing peculiar occurred during the operation. It was of great difficulty, very long, and left the patient much depressed. The sheath of the great vessels was denuded at one point, and the brachial plexus of nerves exposed. The patient, however, recovered; the wound cicatrized, and for about four weeks some relief was experienced, but, at the end of that time, the pain returned; there was an inflammation of the throat, attended with great difficulty of swallowing.

He died with these symptoms, without any external appearance of return of disease, about eight weeks after the operation.

## Figures and Tables

**Figure f1:**
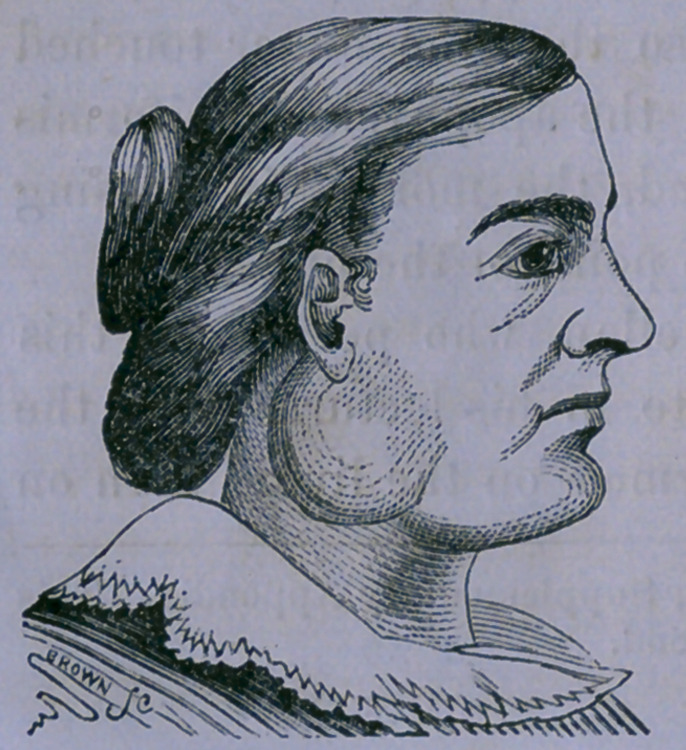


**Figure f2:**
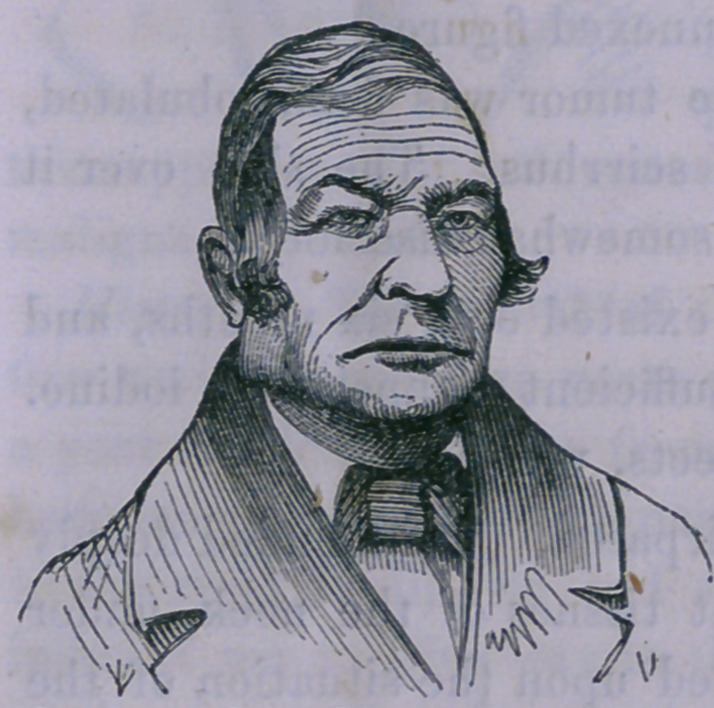


**Figure f3:**
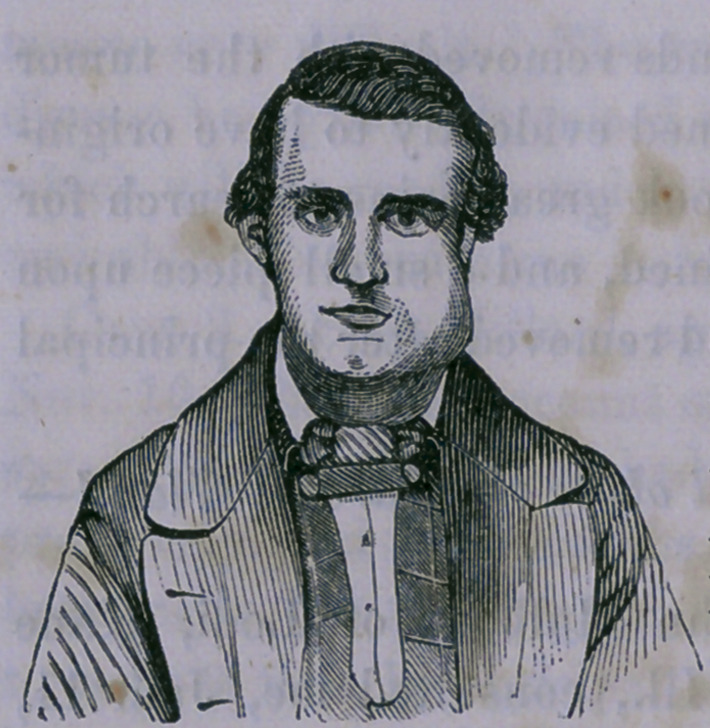


**Figure f4:**